# Supplementary material for: Amotl2a interacts with the Hippo effector Yap1 and the Wnt/β-catenin effector Lef1 to control tissue size in zebrafish
Source: eLife. 2015 Sep 3;4:e08201. doi: 10.7554/eLife.08201 (PMC4596637; doi:10.7554/eLife.08201)
Supplement: Supplementary file 1. — Supplementary tables A, B, C, D related to the ‘Materials and methods’ section. DOI: http://dx.doi.org/10.7554/eLife.08201.042 [file elife08201s022.docx]

**Supplementary file 1A. Primer sequences for generating ISH probes**

| **Primers for ISH probes** | **Sequence** |
| --- | --- |
| amotl2a_1534_fwd | 5’- TGGAGAAGGTGGAAAGGATG- 3’ |
| amotl2a_CB308_rev | 5’- GCAGTTGCAATGGCATACCTA- 3’ |
| wwtr1_7_fwd | 5’- CTTTTCAACTCGGCTGAAACTA- 3’ |
| wwtr1_1398_rev | 5’- TGAGAGCACAAATCCGACAG- 3’ |
| yap1_2526_fwd | 5’- CCCTAGCCTCCTTGTGTGTT- 3’ |
| yap1_3921_rev | 5’- TTCCAGGCCAATTTTAAAGTAA- 3’ |

**Supplementary File1B. Primer sequences for amplifying using zebrafish cDNA as a template**

| **Primers** | **Sequence** |
| --- | --- |
| amotl2a_EcoRI_fwd | 5’- GAATTCTATGAGAACGGCAGAGGAATCATCAG - 3’ |
| amotl2aSTOP_XhoI_rev | 5’- CTCGAGTCAGATGAGGATCTCCACCATGTCA - 3’ |
| amotl2a_BamHI_rev | 5’- GGATCCCTGATGAGGATCTCCACCATGTCA - 3’ |
| amotl2a-MoBS_BamHI_fwd | 5’- GATCCGCCACCATGAGAACGGCAGAGGAATCATCAGC - 3’ |
| amotl2a-MoBS_NcoI_rev | 5’- CATGGTGATGATTCCTCTGCCGTTCTCATGGTGGCG – 3’ |
| amotl2a-MoRes_EcoRI_fwd | 5’- GAATTCTATGCGTACCGCTGAAGAGAGCTCAGGAACGGTGCTGCACC - 3’ |
| yap1_EcoRI_fwd | 5’- GAATTCGATGGATCCGAACCAGCACAAC - 3’ |
| yap1_XhoI_rev | 5’- CTCGAGCTATAGCCAGGTTAGAAAGTTCTCCTT - 3’ |
| wwtr1_EcoRI_fwd | 5’- GAATTCGATGAGCGGTAATCCTCTCCAGC - 3’ |
| wwtr1_XhoI_rev | 5’- CTCGAGCTAGAGCCAGGTGAGGAAGGG - 3’ |

**Supplementary File1C. Plasmid vectors for cloning PCR products**

| **Forward primer** | **Reverse Primer** | **Cloned into** |
| --- | --- | --- |
| amotl2a-MoRes_EcoRI_fwd | amotl2aSTOP_XhoI_rev | pCS2 |
| amotl2a_EcoRI_fwd | amotl2a_BamHI_rev | pCS2-TdT |
| amotl2a-MoBS_BamHI_fwd | amotl2a-MoBS_NcoI_rev | pCS2-gfp |
| amotl2a_EcoRI_fwd | amotl2aSTOP_XhoI_rev | pGBKT7 |
| yap1_EcoRI_fwd | yap1_XhoI_rev | pGADT7 |
| wwtr1_EcoRI_fwd | wwtr1_XhoI_rev | pGADT7 |

**Supplementary File1D. Sequences of the morpholinos injected**

| **Morpholino** | **Sequence** | **Quantity injected** | **Reference** |
| --- | --- | --- | --- |
| Amotl2aMo | 5’-CTGATGATTCCTCTGCCGTTCTCAT-3’ | 1,5ng + p53 | (5) |
| Yap1Mo | 5’-AGCAACATTAACAACTCACTTTAGG-3’ | 11.3ng | (6) |
| TazMo | 5’-CTGGAGAGGATTACCGCTCATGGTC-3’ | 5,9ng + p53 | (7) |
| p53Mo | 5’-GCGCCATTGCTTTGCAAGAATTG-3’ | 1,5x concentration of Mo of interest | (8) |
| Lef1Mo | 5’-CTCCACCTGACAACTGCGGCATTTC-3’ | 2.25ng | (9) |

**Supplementary File1E. Primer sequences surrounding TALEN binding sites**

| **Primers for TALEN PCR** | **Sequence** |
| --- | --- |
| amotl2a_tal2_e3_fwd | 5’ - GGAGTACCCCTTCATGGTCA – 3’ |
| amotl2a_tal2_e3_rev | 5’ - CATCCATGGCAAAATGTGAG – 3’ |
| yap1_tal2_e2_fwd | 5’ - TGGAGGTCAGTGGTTACAGC – 3’ |
| yap1_tal2_e2_rev | 5’ - AGCGGCATGTCATCAGGTAT – 3’ |
